# Genetic Heterogeneity of *HER2* Amplification and Telomere Shortening in Papillary Thyroid Carcinoma

**DOI:** 10.3390/ijms17101759

**Published:** 2016-10-21

**Authors:** Paola Caria, Silvia Cantara, Daniela Virginia Frau, Furio Pacini, Roberta Vanni, Tinuccia Dettori

**Affiliations:** 1 Department of Biomedical Sciences, University of Cagliari, Cittadella Universitaria, Monserrato 09042, Italy; paola.caria@unica.it (P.C.); dvfrau@unica.it (D.V.F.); dettorit@unica.it (T.D.); 2 Department of Medical, Surgical and Neurological Sciences, University of Siena, Siena 53100, Italy; cantara@unisi.it (S.C.); pacini8@unisi.it (F.P.)

**Keywords:** papillary thyroid carcinoma, *HER2* (*Human Epidermal Growth Factor Receptor 2*), Telomere, FISH (fluorescence in situ hybridization)

## Abstract

Extensive research is dedicated to understanding if sporadic and familial papillary thyroid carcinoma are distinct biological entities. We have previously demonstrated that familial papillary thyroid cancer (fPTC) cells exhibit short relative telomere length (RTL) in both blood and tissues and that these features may be associated with chromosome instability. Here, we investigated the frequency of *HER2* (*Human Epidermal Growth Factor Receptor 2*) amplification, and other recently reported genetic alterations in sporadic PTC (sPTC) and fPTC, and assessed correlations with RTL and *BRAF* mutational status. We analyzed *HER2* gene amplification and the integrity of *ALK*, *ETV6*, *RET*, and *BRAF* genes by fluorescence in situ hybridization in isolated nuclei and paraffin-embedded formalin-fixed sections of 13 fPTC and 18 sPTC patients. We analyzed *BRAF^V600E^* mutation and RTL by qRT-PCR. Significant *HER2* amplification (*p* = 0.0076), which was restricted to scattered groups of cells, was found in fPTC samples. *HER2* amplification in fPTCs was invariably associated with *BRAF^V600E^* mutation. RTL was shorter in fPTCs than sPTCs (*p* < 0.001). No rearrangements of other tested genes were observed. These findings suggest that the association of *HER2* amplification with *BRAF^V600E^* mutation and telomere shortening may represent a marker of tumor aggressiveness, and, in refractory thyroid cancer, may warrant exploration as a site for targeted therapy.

## 1. Introduction

The most common histological subtype of non-medullary thyroid carcinoma (NMTC) is papillary thyroid carcinoma (PTC), which represents 75%–85% of all thyroid cancer. Although mostly sporadic (sPTC), there is some evidence for a familial form of PTC (fPTC) not associated with known Mendelian syndromes. Familial PTC is observed in approximately 5%–10% of NMTC cases [1] and, despite the extensive research dedicated to understand if it is a distinct biological entity than sPTC [2], this distinction remains controversial. Indeed, the most common somatic alterations, such as mutations in *RAS* and *BRAF* and rearrangements of *RET/PTC* and *NTRK1*, exhibit similar prevalence and distribution in both sPTC and fPTC [3]. No oncogenic germline mutations of these genes have been detected in fPTC cases [4]. However, there is an ongoing debate on the possible association of *HAPB2* germline mutation to the predisposition to familial forms of NMTC [5,6,7,8]. Generally speaking, most PTC can be treated effectively with surgery and radioactive iodine therapy. However, for cases in which these treatments are not effective, targeted drugs might be considered. Kinase inhibitors, such as sorafenib and lenvatinib, are now used as targeted drugs [9,10]. Less frequent genetic alterations, such as rearrangements of *ALK* [11] and *ETV6* [12], identified in sPTC, but not yet investigated in fPTC, also represent new therapeutic targets. In addition, the amplification of the *HER2* gene, reported in highly-malignant PTC nodules [13], might be added to the list of drug-targetable genes. The *HER2* amplification in PTC was observed by fluorescence in situ hybridization (FISH), and average telomere length in *HER2-*positive (*HER2+*) PTC was significantly shorter than *HER2-*negative (*HER2−*) PTC [13]. Of possible significance, shorter average telomere length has also been reported in fPTC compared to sPTC [14]. These observations prompted us to verify *HER2* amplification and telomere length status in 13 fPTC and 18 sPTC. Tumors were also investigated for integrity of the *RET* gene, which is rearranged in 10%–40% of PTC, and *ALK*, *ETV6*, and *BRAF* genes, which are rearranged in a minority of PTC. *BRAF^V600E^* mutation, which is associated with an aggressive biological behavior [15], was also evaluated. Our results indicate an increased prevalence of occasional *HER2* gene intermediate amplification in fPTC compared to sPTC, and shorter telomeres in all fPTC, including those with *HER2* amplification, compared to sPTC. In addition, all *HER2+* samples invariably possessed the *BRAF^V600E^* mutation, but not vice versa. The association might represent a marker of tumor aggressiveness and, in refractory thyroid cancer, may indicate possible exploration for targeted therapy. Additionally, the simultaneous occurrence of these three specific molecular alterations may be suggestive of the existence of a specific fPTC subgroup.

## 2. Results

### 2.1. Human Epidermal Growth Factor Receptor 2 (HER2) Amplification in Familial Papillary Thyroid Carcinoma (fPTC) and Sporadic Papillary Thyroid Carcinoma (sPTC)

**Figure 1:**
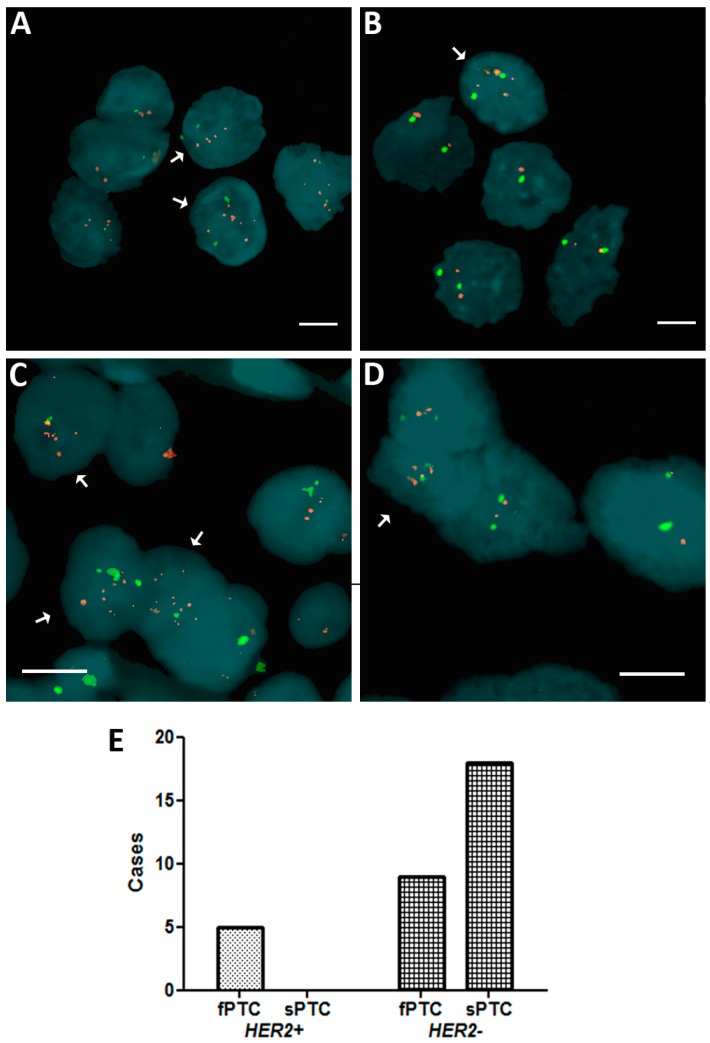
Distribution of *HER2* (*human epidermal growth factor receptor 2*) amplification in familial papillary thyroid carcinoma (fPTC) and sporadic papillary thyroid carcinoma (sPTC) nuclei. Arrows point to isolated nuclei with extra copies of the *HER2* gene (**red spots**) in the presence of disomy 17 (chromosome 17 centromere-specific alphoid repetitive DNA, **green spots**) in fPTC (**A**); and sPTC (**B**); and in nuclei in formalin-fixed paraffin-embedded (FFPE) sections of fPTC (**C**); and sPTC (**D**). The distribution of the amplification in FFPE sections of fPTC versus sPTC was significant (*p* = 0.0076) (Fisher exact test) and indicative of genetic heterogeneity heterogeneity according to Vance criteria [17] (**E**). Scare bar = 10 µm.

*HER2* amplification was evaluated in 13 fPTC (seven females, mean age at diagnosis of 53.7 ± 14.2; six males, mean age at diagnosis 49.0 ± 21.5) and 18 sPTC (15 females, mean age at diagnosis 46.6 ± 7.8; three males, mean age at diagnosis 43.6 ± 13.6). We found that isolated fPTC and sPTC nuclei were *HER2−* according to the Wolff criteria (originally developed for breast cancer formalin-fixed paraffin-embedded (FFPE) examination) [16], although a number of cases showed scattered *HER2+* cells, ranging from 1.4% to 9% in fPTC and from 1% to 2.4% in sPTC (Figure 1A,B). Based on these observations, and on the lack of specific criteria for the evaluation of *HER2* amplification in thyroid tumors, we analyzed the distribution of HER2+ cells in these cases to determine if they met the criteria for the presence of genetic heterogeneity (>5% and <50%) according to Vance [17]. We found significant genetic heterogeneity in the distribution of *HER2* amplification in fPTC compared to sPTC (*p* = 0.0076) (Figure 1C–E). We found that 5/13 (38.5%) fPTC cases showed 5.1% to 10% HER2*+* cells. FISH on FFPE from sPTC confirmed the findings obtained from isolated nuclei that no sPTC case exceeded the cut-off value. When fPTCs were analyzed by immunohistochemistry using an anti-c-erbB2 antibody [18] to detect HER2 protein expression, inconsistent results compared to the FISH analysis were obtained. However, this result was possibly biased by the age of the available histological sections (7–20 years).

### 2.2. Rearrangements of ALK, BRAF, ETV6, and RET Genes

**Figure 2:**
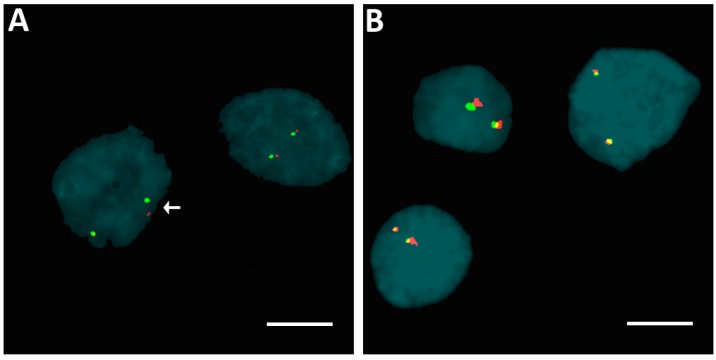
Examples of fluorescence in situ hybridization (FISH) in isolated nuclei for the identification of genes specifically rearranged in papillary thyroid carcinoma (PTC). Arrows point to the split of the red/green signal of a *RET* break-apart [19] probe in the case of sPTC, indicating broken *RET* (**A**); and to un-split red/green signals of an *ALK* break-apart probe in the case of fPTC, indicating unbroken *ALK* (**B**). **Red spot**: 300 kb probe DNA fragment; **Green spot**: 442 kb probe DNA fragment; Scare bar = 10 µm.

We found no rearrangements of *ALK*, *BRAF*, and *ETV6* genes by fluorescence in situ hybridization. We found one case of sPTC with a *RET* rearrangement, the remaining cases exhibited neither disruptions nor numerical changes in *RET* gene (see examples in Figure 2A,B).

### 2.3. Telomere Length and BRAF^V600E^ Mutation in fPTC and sPTC Patients

**Figure 3:**
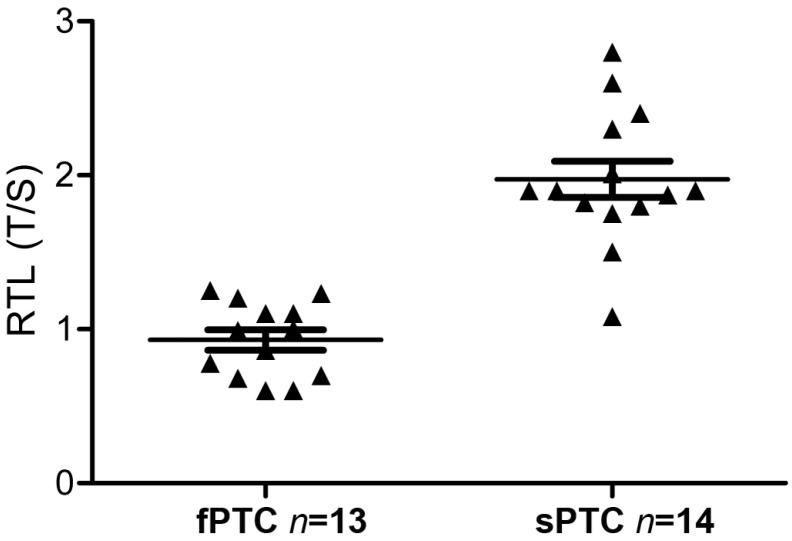
fPTC and sPTC relative telomere length (RTL). RTL was measured by q-PCR, and was expressed as the ratio (T/S) of the telomere (T) repeat copy number to a single-copy gene (S). The difference in RLT between fPTC and sPTC samples was significant (*p* < 0.001) (Mann-Whitney *U*-test). Triangles represent the RTL of each case; the upper and lower lines represent the interquartile range of the distribution (25th–75th percentile); the middle line represents the median.

Relative telomere length was significantly shorter in fPTC samples than in sPTC samples: median = 0.93 (25th–75th percentile: 0.6–1.2) vs. 1.9 (25th–75th percentile: 1.8–2.3) for fPTC vs. sPTC, respectively (*p* < 0.001) (Figure 3). This result was not due to a difference in the patient’s age and sex, as sporadic cases were selected to be age/sex-matched with familial patients (Table 1).

**Figure 4:**
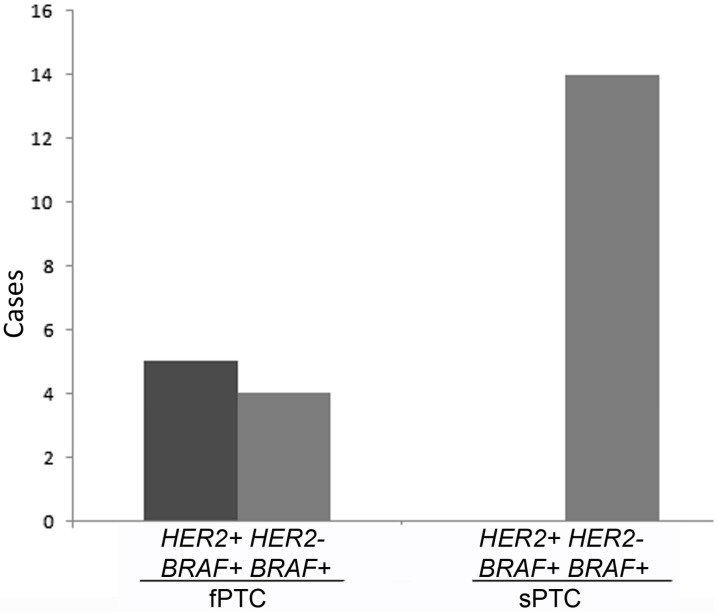
Distribution of *HER2* amplification and *BRAF^V600E^* mutation in fPTC and sPTC tumors.

*BRAF^V600E^* mutation was detected in 9/13 (69.0%) fPTC and 14/18 (78%) sPTC (*p* = 0.68), which was not statistically significant. All *HER2+* fPTC were *BRAF^V600E^*-positive (*BRAF*+), although not all *BRAF*+ fPTC were *HER2*+ (Figure 4).

## 3. Discussion

Amplification of the *HER2* gene in thyroid cancer was first uncovered by FISH analysis of follicular cells from highly malignant PTC nodules [13]. They observed that *HER2+* PTC exhibited shorter telomeres than *HER2*− PTC. PTC is an entity mostly recognized as sporadic, although the familial form may account for approximately 5% of cases [1,2]. Familial PTC may occur in combination with other Mendelian cancer syndromes (familial adenomatous polyposis, Gardner’s syndrome, Peutz-Jeghers syndrome, and Cowden’s syndrome) or may be unassociated with other neoplasms in familial aggregates. However, although the risk of developing extra-thyroidal malignancy in non-Cowden’s syndrome is documented [21,22], the clinical correlation between sporadic breast cancer (20% of which are *HER2+*) [23] and PTC is still controversial [24,25], and the co-occurrence of both disorders in the same individual is a subject of extensive debate [26,27]. None of our fPTC patients had clinical or pathological evidence of hereditary syndromes associated with NMTC, breast cancer, or other types of sporadic tumors, except for one male patient who had a previous squamous cell carcinoma of the auricle. The thyroid cancer of this patient was not associated with any genetic alterations of the genes examined here. As more than 5%, but fewer than 50%, of nuclei were found to be amplified in our FFPE sections, we use the Vance criteria [17] in the interpretation of our results. Our data indicated a significant difference in *HER2* amplification (*p* = 0.019) in fPTC compared to sPTC. This finding indicates a degree of genetic heterogeneity in the fPTC group and suggests that *HER2*+ cells in fPTC possibly undergo apoptosis to a lesser extent than in sPTC. The observation adds thyroid carcinoma to the list of tumors that exhibit *HER2*+. *HER2*+ is indeed observed in a growing number of other tumors, including advanced gastric and esophageal cancer [28], ovarian [29], colon [30], bladder, lung, uterine, cervix, head and neck, and endometrial cancer [31]. The extracellular domain of the HER2 receptor has an essential role in cell proliferation and anti-apoptotic processes, making *HER2+* breast and gastric/gastroesophageal cancers more likely to respond to targeted therapies in combination with chemotherapy than *HER2−* tumors. A wide range of solid tumors showing deregulation of *HER2* expression are regarded as biologically aggressive. Familial PTC is often associated with a more severe phenotype than its sporadic counterpart [32], and often harbor *BRAF^V600E^* mutation. *BRAF^V600E^* is considered to have a prognostic value in PTC [33], and usually identifies differentiated thyroid tumors with advanced clinicopathological features. *BRAF^V600E^* is also strongly associated with PTC patient mortality [15]. In contrast to lung adenocarcinoma, in which *HER2* amplification and *BRAF^V600E^* mutation appear to be mutually exclusive events [34], here we found that all *HER2*+ fPTC bore the *BRAF^V600E^* mutation, although not all *BRAF^V600E^*-positive nodules had *HER2* amplification. Of significance, none of the *BRAF^V600E^*-positive sPTC were *HER2*+, despite the high frequency of *BRAF^V600^* mutations in this cohort. It is not entirely clear how this discrepancy should be interpreted, considering the limited size of our cohorts. In addition, we do not know whether *HER2* amplification and *BRAF^V600E^* mutation coexist in the same cells within a tumor or if they are segregated in different clones. We do not know, either, if the condition is different in our *HER2+* fPTC versus sPTC with *HER2+* cells <5%.

The other evaluated genes, *ALK*, *BRAF*, *ETV6*, and *RET*, exhibited extensive integrity. The only case bearing a *RET* disruption was *BRAF^V600E^*-negative. Moreover, we found a significant difference (*p* < 0.001) of RTL in fPTC nodules compared to sPTC nodules, in agreement with our previous investigations [14,35]. Telomere length regulation plays a crucial role in genome instability and tumorigenesis [36]. Dysfunctional telomeres can increase chromosome instability by causing either fusion of chromosomes or fusion of sister chromatids, bringing the formation of anaphase bridges and the beginning of the so-called breakage-bridge-fusion cycles [37]. Although biased by the small number of patients investigated in our two cohorts (forced by the low frequency of fPTC), our data stigmatize significantly shorter RTL in fPTC cells versus sPTC cells. This result is in line with the reported predisposition of fPTC patients toward spontaneous chromosome fragility [35]. This observation poses the basis for further investigation exploring the existence of a possible specific three-dimensional (3D) altered telomere organization in fPTC. Telomere remodeling is a feature of cancer cells [38] and may identify tumor subgroups [39,40]. On the other hand, alterations in the telomere 3D profile have been reported in a murine model of thyroid tumors [41]. Recurrent somatic mutations in the promoter of TERT, the catalytic subunit of the enzyme telomerase, has been reported in PTC [42], often in concomitance with mutated *BRAF* [43]. A subclonal distribution in the rare PTC that harbor the alteration, in contrast to a clonal distribution in the poorly-differentiated and anaplastic tumors has been observed [44]. Unfortunately, the scanty material available from our cases prevented the possibility to establish a putative association of TERT promoter mutations with telomere length in our PTCs.

Our finding on RTL are, substantially, in keeping with the Sugishita data [13]. However, in contrast, 38.5% of our fPTC, but none of our sPTC, showed *HER2* amplification, indicating an apparently preferential association with fPTC. In this regard, the small number of patients investigated in our two cohorts might constitute a bias. Nevertheless, as a whole, the finding of *BRAF^V600E^* mutation in association with *HER2*+ genetic heterogeneity, short telomere length, and prevalence of multifocal tumors seems to not be a rare molecular event in fPTC and may characterize a subgroup of fPTC. The response of refractory fPTC patients of this subgroup to target therapy of trastuzumab and lapatinib should be explored.

## 4. Materials and Methods

### 4.1. Sample Collection

The PTCs (13 fPTC and 18 sPTC) considered in the present study were selected from the pathological files of the University of Siena. Familial recurrence of the disease was defined as the presence of at least one first-degree relative with differentiated thyroid carcinoma in the absence of any other familial syndrome. None of the fPTC patients presented with any other sporadic tumor, including breast cancer, except for one male patient with a previous squamous cell carcinoma of the auricle. The histology of all tumor samples was classified according to the World Health Organization guidelines [20]. The fPTC cases were selected from 13 families, randomly choosing one affected subject from each family (the oldest affected subject): seven females (mean age at diagnosis of 53.7 ± 14.2, with an age range of 29–69 years) and six males (mean age at diagnosis of 49.0 ± 21.5, range 24–81). Four fPTC tumors were classified as follicular variants and nine were classical PTC. Seven out of the 13 families (53.8%) had three or more affected members, two out of 13 (15.3%) had two members with PTC and at least three members operated for multi-nodular goiter, and four out of 13 had only two members affected by thyroid cancer. In these cases, the phenomenon of genetic anticipation was observed with the second generation acquiring the disease at an earlier age and having more advanced disease at presentation.

The sPTC cases were from 15 females (mean age at diagnosis of 46.6 ± 7.8, range 31–64) and three males (mean age at diagnosis 43.6 ± 13.6, range 31–58). Fourteen were classified as classical PTC, two as follicular variants, one as a diffuse sclerosing variant, and one as a trabecular variant (Table 1). Informed consent was obtained from each patient after a full explanation of the purpose and nature of all of the procedures to be used. All data from the patients were handled in accordance with local ethical committee-approved protocols and in compliance with the Helsinki declaration

### 4.2. Fluorescence In Situ Hybridization

Thick sections (30 µm) were obtained from formalin-fixed paraffin-embedded (FFPE) tissue blocks of the thyroid nodules or, to assess probe cut-off, from the apparently tumor-free tissue of the contralateral lobe. Nuclei were isolated as reported [45], and were investigated by FISH, using specific probes and a standard protocol [46].

### 4.3. Detection of HER2 Gene Copy Number Alterations or Amplification

*HER2* amplification was determined by counting the total numbers of *HER2* and CEP17 (chromosome 17 centromere-specific alphoid repetitive DNA, Abbott Molecular, Abbott Park, IL, USA) signals per nucleus with a mean of 89.5 (range 34–175) nuclei. The ratio of *HER2* signals to CEP17 (centromeric probe for chromosome 17) signals was calculated according to ASCO/CAP (American Society of Clinical Oncology/College of American Pathologists Guideline) criteria refined for breast cancer [16].

Nodules with, or suspected to have, *HER2* amplification were then re-evaluated by FISH on 4 µm histological sections to assess the distribution of possible clones. The tumor area, marked by the pathologist [47], was entirely scored. As more than 5%, but fewer than 50%, of nuclei were found to be amplified, the Vance criteria were used to define the distribution of abnormal cells [17].

Consecutive histologic sections were used to assess *HER2* expression by immunohistochemistry. The expression of HER2 protein was determined using anti-c-erbB2 antibody (Dako, Glostrup, Denmark) in accordance with the manufacturer’s instructions.

Detection of *ALK*, *BRAF*, *ETV6*, and *RET* rearrangements. Commercially available break-apart or single gene probes for *ALK*, *BRAF* (Abbott Molecular, Abbott Park, IL, USA), and *ETV6* (Kreatech Diagnostic, Amsterdam, The Netherlands) were used to verify gene integrity in nuclei isolated from FFPE tissue blocks. An arbitrary cut-off of 3% was employed, as control cells showed no split signal in 200 scored nuclei per sample. For the *RET* gene, a homebrew probe and a previously described cut-off value were used [19]. Microscopic analysis was performed with an Olympus BX41 epifluorescence microscope and a charge-coupled device camera (Cohu, San Diego, CA, USA) interfaced with the CytoVision system (software version 3.9; Applied Imaging, Pittsburg, PA, USA).

### 4.4. Telomere Length and BRAF^V600E^ Mutation

DNA was extracted from fresh or FFPE tissues, using the QIAamp^®^ DNA Mini Kit (Qiagen, Milano, Italy) following the manufacturer’s instructions. RTL of sPTC was determined by quantitative PCR, carried out on 30 ng/μL genomic DNA using an MJ Mini Personal Thermal cycler (Bio-Rad, Milano, Italy) as described [14]. RTL values of the fPTC examined in the present study were reported previously [14]. Relative telomere length was calculated as the ratio of telomere repeats to a single-copy gene in experimental samples using standard curves. This ratio is proportional to the average telomere length. The *36B4* gene, which encodes acidic ribosomal phosphoprotein P0, was used as the single-copy gene [14]. For analysis of the *BRAF^V600E^* mutation, DNA was amplified in a final volume of 50 µL of 2× PCR Master Mix (AmpliTaq Gold^®^ PCR Master Mix, Applied Biosystems, Milano, Italy) and a final primer concentration of 200 nM. Primer sequences, PCR conditions, and interpretation of results were as previously described [48].

### 4.5. Statistical Analyses

Mann-Whitney *U*-test (IBM SPSS Statistic version 2.1 software, Armonk, NY, USA) was used for statistical analysis of differences in RTL. Fisher exact test was used to compare *HER2* gene amplification. All *p-*values were two-sided and *p* less than 0.05 was considered significant.

Epidemiological data are presented as the mean ± SD and median when necessary. The *t*-test for independent data was performed for normal variables. To evaluate significant differences in data frequency we analyzed 2 × 2 contingency tables by the Fisher exact test. Tables with sizes larger than 2 × 2 were examined by the Chi-squared test.

## Figures and Tables

**Table 1:** Characteristics of patients.

Tumor	Age at Diagnosis (Mean ± SD)	Sex (Males %)	PTC Size (Median/IQR)	TNM	Extrathyroidal Invasion *N* (%)	Multifocality *N* (%)	Lymphonode Metastases at Diagnosis *N* (%)	Final Outcome * *N* (%)	Follow-up (Mean Years)	Histology
*fPTC* (*n = 13*)	51.5 ± 17.0	6 (46.1%)	11/11.5	*pT1* 8 (61.5%) *pT*2 2 (11.7%) *pT3* 3 (23.0%)	5 (38.5%)	7 (53.8%)	3 (23%)	*Remission* 9 (69.2%) *Persistent disease* 4 (30.8%)	7.59 ± 3.9	9 CV-PTC 4 FV-PTC
*sPTC* (*n = 18*)	46.1 ± 8.5	3 (16.6%)	9.5/8.5	*pT1* 8 (44.4%) *pT2* 1 (5.5%) *pT3* 9 (50.%)	9 (50%)	6 (33.3%)	5 (27.7%)	*Remission* 11 (61.1%) *Persistent disease* 2 (11.1%)	5.5 ± 2.8	14 CV-PTC 2 FV-PTC 1 SV-PTC 1 TR-PTC

CV-PTC—classical variant of papillary thyroid carcinoma; fPTC—familial papillary thyroid carcinoma; FV-PTC—follicular variant of papillary thyroid carcinoma; IQR—Inter Quartile Range; *N*—number of cases; PTC—papillary thyroid carcinoma; sPTC—sporadic papillary thyroid carcinoma; SV-PTC—sclerosing variant of papillary thyroid carcinoma; TNM—(Tumor (limph) Node Metastasis) classification [20]; TR-PTC: trabecular variant of papillary thyroid carcinoma; *—six patients were lost to follow-up.
